# FV Vectors as Alternative Gene Vehicles for Gene Transfer in HSCs

**DOI:** 10.3390/v12030332

**Published:** 2020-03-19

**Authors:** Emmanouil Simantirakis, Ioannis Tsironis, George Vassilopoulos

**Affiliations:** 1 Gene Therapy Lab, Biomedical Research Foundation of the Academy of Athens, Division of Genetics and Gene Therapy, Basic Research II, 11527 Athens, Greece; esimantirakis@bioacademy.gr (E.S.); tsironis@upatras.gr (I.T.); 2 Division of Hematology, University of Thessaly Medical School, 41500 Larissa, Greece

**Keywords:** foamy virus, gene therapy, HSC, gene marking, FV gene transfer to HSCs, gene therapy alternatives

## Abstract

Hematopoietic Stem Cells (HSCs) are a unique population of cells, capable of reconstituting the blood system of an organism through orchestrated self-renewal and differentiation. They play a pivotal role in stem cell therapies, both autologous and allogeneic. In the field of gene and cell therapy, HSCs, genetically modified or otherwise, are used to alleviate or correct a genetic defect. In this concise review, we discuss the use of SFVpsc_huHSRV.13, formerly known as Prototype Foamy Viral (PFV or FV) vectors, as vehicles for gene delivery in HSCs. We present the properties of the FV vectors that make them ideal for HSC delivery vehicles, we review their record in HSC gene marking studies and their potential as therapeutic vectors for monogenic disorders in preclinical animal models. FVs are a safe and efficient tool for delivering genes in HSCs compared to other retroviral gene delivery systems. Novel technological advancements in their production and purification in closed systems, have allowed their production under cGMP compliant conditions. It may only be a matter of time before they find their way into the clinic.

## 1. Introduction

Hematopoietic Stem Cells (HSCs) are rare cells residing in the Bone Marrow (BM) that can support blood cell production for the lifetime of an individual. This is achieved through HSCs potential to differentiate and self-renew [1]. The self-renewal process is the central dogma in stem cell biology and is a character that is lost when HSCs are extensively manipulated ex vivo; this observation argues for the substantial contribution of the BM milieu in supporting HSCs. Practically, both HSC nature and BM nurture, in a coordinated interplay, allow HSCs to express their unique characters [2]. What has been elusive is the HSC per se; HSC cannot be identified by morphology but only by surface markers and functional assays.

In humans, HSCs are considered as CD34+ cells residing in the mononuclear cell population of the BM. Not all HSCs are CD34+ but a significant fraction of CD34+ cells are HSCs; this circular argument can be rephrased to convey that CD34+ cells are enriched in HSC and CD34 marking is used to assay whether a certain cell population has enough HSC to serve as a donor for a transplantation experiment [3]. The latter is the sole surrogate marker to assay whether a population of cells has the potential to support hematopoiesis in a myeloablated host. In addition, Stem Cell Transplantation (SCT), is the ultimate marker to support the potential of a viral vector to correct a genetic defect in a HSC; the marked or corrected cells of the donor will be present in the blood cells of the recipient host and will provide phenotypic correction for a lifetime.

Allogeneic SCT (ASCT) has been used since around the ‘70s for the treatment of acute leukemias. ASCT for genetic defects such as thalassemia was introduced in the 90s and achieved high cure rates for those who had a suitable donor [4]. In contrast, transplantation with autologous gene-corrected HSCs entered the therapeutic armamentarium around the year 2000 and was only recently endorsed as a therapeutic option by the FDA [5]. Gene correction for HSC requires the permanent integration of the vector in the host-HSC genome; if episomal, the vector is destined to dilute out at some point in the subsequent cell divisions since episomes do not faithfully follow the cell’s DNA mitotic cycles [6]. Retroviruses are viruses with potential of permanent integration, which is a prerequisite for viral gene expression and a productive life cycle. 

There are two subfamilies of Retroviruses: the first is the *Orthoretrovirinae* that includes the Genera *Lentivirus* and *Gammaretrovirus* (GV), and the second is *Spumaretrovirinae* that includes the genera *Simiispumavirus* and *Felispumavirus* [7]. The human foamy virus isolates are actually chimpanzee foamy viruses. There are three to date: human isolate HSRV clone 13 SFVpsc_huHSRV.13, human isolate BAD327 SFVptr_huBAD327, and human isolate AG15 SFVptr_huAG15 [7]. For simplicity and familiarity reasons, in this article, wherever we refer to SFVpsc_huHSRV.13 we shall use the name PFV, HSRV, or FV vectors. The first vectors to be developed for gene transfer purposes were those with the gamma-retroviral backbones and were used for genetic correction of immunodeficiency syndromes after extended preclinical validations [8,9]. However, along with the successes, novel problems emerged; The not-so-random insertion of the retroviruses close to oncogenes, resulted in the development of leukemias [10]. The development of high throughput technologies allowed the mapping of Retroviral Integration Sites (RIS) and shed light on the gammaretroviral vector potential to cause leukemias, in relation to their preference for specific genomic sites. Gammaretroviral vectors as well as Lentiviral vectors demonstrate a preference for the integration of their retroviral cDNA into transcriptionally active sites in the host genome [11]. In addition, the Gammaretroviral vectors showed an increased preference for landing near oncogenes, a potentially dangerous condition [12]. Further data showed that different genera of retroviruses have distinct preferences for genomic integrations: Gammaretroviral vectors and Foamy Viral vectors prefer to integrate in proximity to transcription start sites and regulatory elements like CpG islands, while Lentiviral vectors tend to integrate within coding sequences [13,14,15]. Beyond safety, a problem that retroviruses had in relation to the need for long-term expression in the context of HSCs, was the frequent observed silencing of the transferred transgene. Although silencing was related to the methylation of the retroviral DNA [16,17], a problem that could be solved with the use of insulator elements [18] or partially reversed with hypomethylating agents [19], the quest for a more efficient gene transfer vehicle did not seize. 

In a chronological order, the two other kinds of retroviruses that were tested for their HSC gene transfer potential were the Lenti- and the Foamy- or Spuma- derived vectors. In a seminal paper on Lenti-vectors, the authors reported long-term gene marking in CD34+ cells transplanted in NOD/SCID mice [20]. Reports on the potential of Spuma- (or Foamy Virus, FV) derived vectors to transduce efficiently both murine and human HSCs and to express the transferred gene in a long-term manner, came soon after [21]. Although, currently, Lenti has gained tremendous popularity as a gene transfer tool and has obtained commercial approval for human use, FV-derived vectors are a safe and efficient alternative for gene transfer into HSCs [22,23]. Beyond HSC gene transfer, FV vectors of feline origin have been developed as gene transfer vehicles and have been tested so far in cell lines [24]; an interesting application of these vectors is their use as vehicles for the transfer of genes that induce immune responses to feline viruses [25]. In this review, we will present all the relative data that confirm the potential of FV-derived vectors as a non-inferior vehicle for HSC gene transfer and therapy. The main features of each retroviral gene delivery system are summarized in Table 1.

## 2. Features of FV Vectors for HSC Gene Delivery

A number of features are essential for any vector to be suitable for HSC gene transfer. Target cell tropism is the principal condition, and FV vectors have shown that they can transduce a number of cell lines and murine hematopoietic progenitors and stem cells early on during the vector development history [21,33,34]. The broad spectrum of permissive cells for FV transduction, suggested that an abundant molecule on the cell surface was facilitating FV attachment and/or entry. This molecule was later identified as heparan sulfate [35,36]. The expression of heparan sulfate in a wide variety of cells and its use by FVs as a non-specific receptor could explain the wide cell tropism that this vector demonstrates.

Another key principal for any gene transfer vehicle is its ability to deliver its cargo into non-dividing cells such as the HSCs. This principle was assessed through experiments that tested FV vector transgene expression in vivo after transplantation of transduced HSCs. Given that reporter gene expression can be traced in transplant recipients, it can be inferred that true, non-dividing HSCs were transduced by FV vectors [21,37]. However, gene transfer in HSCs occurs after the 5FU treatment of the HSC donor animals and the ex vivo manipulation of cells under strong cytokine stimulation; in such conditions, HSCs are practically forced to divide. Thus, conclusions on FV gene transfer into non-dividing cells from such experiments could not be confidently reached. It was later shown in a different cellular system that although FV DNA lacks nuclear localization signals, the FV DNA can survive long enough in the cytoplasm in anticipation of a subsequent cell division that will result in nuclear membrane break down [38,39]. Under such conditions, FV DNA can enter the nucleus and establish a productive infection or transduction. However, it should be clarified that FVs are complex retroviruses. Their replication demands an RNA intermediate from which viral cDNA synthesis occurs by the activity of viral reverse transcriptase. Furthermore, in in vitro systems, it has been observed that the reverse transcription of viral RNA is completed before virus budding [13].

Following cellular entry and nuclear penetration, gene transfer vehicles must also have long-term expression in daughter cells. This is a prerequisite for therapeutic procedures whose results are expected to span the lifetime of an individual. Vector gene silencing occurs through DNA methylation/histone acetylation and has been a central problem in gene transfer with retroviral vectors [16,17]. This does not seem to be a problem with FV vectors, since (i) transgene silencing has not been reported in any of the in vivo studies published so far and (ii) it has been shown that FV Long Terminal Repeats (LTRs) have the potential to insulate the vector genome when integrated in the host cell DNA [31,40]. The FV insulation can be seen as a double-edged sword; it protects the transgene from external effects but also protects the genomic environment from the effects of the FV vectors. This prompts the issue of activation of neighboring oncogenes at the integration site. Compared to LVs and GVs in in vitro immortalization assays, FV vectors had the lowest rate of read-through transcripts. When the LMO2 site was targeted for insertion, LMO2 mRNA increments were 280x for GV, 200x for LV, and 45x for FV vectors, normalized for genomic integration site, indicating insulator properties of the LTR. This low read-through transcription of an integrated FV vector occurs due to a 36 bp long CTCF binding motif inside its LTR. CTCF is the major chromatin insulator protein in vertebrates and binds the CCCTC sequence via various combinations of 11 zinc fingers [41].

**Figure 1:**
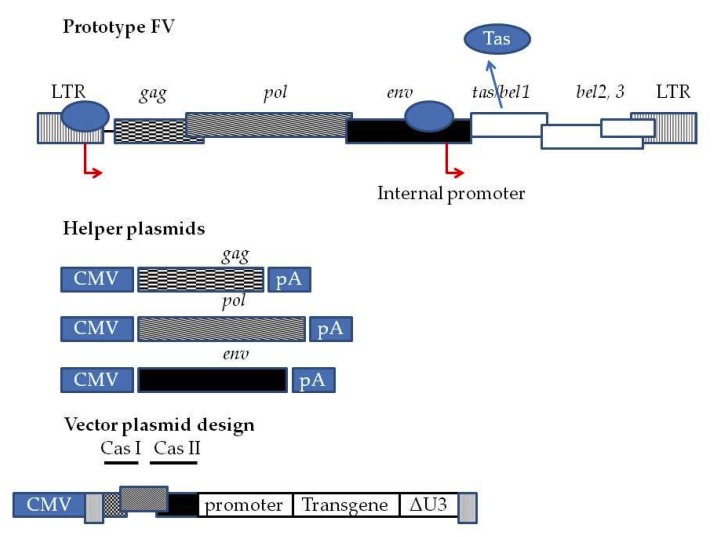
Third generation Foamy Virus (FV) vector system. At the top wtPFV provirus genome is depicted. The LTR contains the entire U3 region of the Human Foamy Virus (HFV.). Red arrows indicated the promoters driving the expression of FV genes, as well as the internal promoter present inside the *env* sequence driving the expression of *tas*. *tas* is expressed at basal levels when the provirus integrates into host cell genome. Upon translation, Tas binds to the LTR and internal promoter and enhances the transcription of *gag*, *pol*, and *env.* Additionally, Tas enhances the transcription of *bel1*, *bel2*, and *bel3. bel1* and *bel2* transcripts splice into a single mRNA whose translation generates Bet. In third -generation systems, the FV genome is split into a four-plasmid system comprised of three helper plasmids encoding *gag*, *pol,* and *env* under the control of a CMV promoter and a pA (polyadenylation signal) to allow the packaging cell lines to express the proteins at high levels. Transfer vector is comprised of deleted LTRs. Viral RNA expression is driven by a CMV promoter and an important cis-acting element sequence between the 5′LTR and *gag*, a part of the 5′ *gag* (CAS I) sequence, a part of the 3′ *pol* (CAS II) sequence and a part of the 5′ *env* sequence. These sequences are necessary for efficient virion assembly. The CMV 5′LTR fusion renders the viral vector production in packaging cell lines (HEK293T) Tas independent. Moreover, there is a deletion of the U3 region of LTR. Following integration into the transduced cell genome, the Tas dependent LTR promoter is regenerated. This fact renders the vector SIN and shuts the expression driven by 5′ LTR off. Transgene expression is driven by internal promoters.

After cell tropism and long-term expression is the issue of safety. From data on zookeepers that are chronic FV carriers, we have reassuring evidence that wtFV, causes no harm to its hosts [42,43]. However, beyond the wtFV non-pathogenic characteristics, foamy viral vectors possess a number of features making them attractive for use in gene therapy. Current FV designs consist of a split four plasmid system: the transfer vector and three accessory or helper packaging plasmids [44,45]. The packaging plasmids encode the Gag, Pol, and Env proteins; the Tas (or Bel1) and Bet proteins required for the infection and replication of the wtFV are dispensable for vector production and can be omitted. Furthermore, the vectors have a self-inactivating (SIN) design; deletion of the 3’LTR U3 region in the transfer plasmid is copied in in the 5’LTR sequence during packaging, rendering the deleted FVs safe for gene therapy applications. Bet deletion also renders the host cell susceptible to superinfection (multiple rounds of infection), a desirable feature for difficult to transduce cell targets [15,31,46]. PFV vectors were developed independently in the States and in Europe by Russel’s [44] and Rethwilm’s [47] groups, respectively. Here, we shall elaborate on the vectors developed by the Russel group. The FV vectors originated from the wtFV strain SFVpsc_huHSRV.13 [48]. The wt provirus map is presented in Figure 1. The viral cDNA contains three overlapping open reading frames (ORFs). The viral cDNA also encodes the genes *gag*, *pol,* and *env*. The latter three are typical of all retroviruses. The aforementioned overlapping ORF encodes the genes *bel1/tas*, *bel2*, and *bel3*. Tas is a transactivator protein that binds into the internal promoter present in *env* and 5′LTR promoters and enhances the transcription of the wt integrated FV viral genome. Additionally, Bet results from the translation of spliced *bel1* and *bel2* ORF mRNA. Bel1-3 and Bet are not required for viral replication in vitro. Thus, they are omitted from vector designs. The current vectors comprise of four plasmids. A transfer vector with deleted viral LTRs. Some important cis-acting elements exist between the 5′LTR and *gag*, a part of the 5′ *gag* (CAS I) sequence, a part of the 3′ *pol* (CAS II) sequence, and a part of the 5′ *env* sequence. The 3′ LTR bears a deletion in the U3 region. Additionally, the 5′ LTR is fused with the CMV promoter to render the vector Tas independent. In order to avoid the generation of the replication competent virus in the packaging cell lines, the Gag, Pol, and Env are expressed by separate plasmids. The coding sequences bear minimal overlap [21,28,44,49].

In the recent years, more light has been shed on the integration site profile of FVs on the host cell genome. On studies performed on human HSCs transduced with FV vectors, the vectors seemed to prefer integrating upstream of transcription start sites specifically within CpG islands, with only 4.4% of the integration sites to be 50kb proximal to proto-oncogenes [13,31,41]. Although Integration Sites (IS) proximity to the genes remains largely random, the preference that the FV vectors display makes them relatively safer than GV and LV vectors, as they prefer constitutively lamina associated regions (cLAD) and less often CpGs [15] to integrate.

An issue of interest in gene therapy applications is the transgene payload that a vector can carry. Transgene payload is of paramount importance because regulated gene expression requires non-coding sequences of significant length. As a rule of thumb, one should not exceed the length of the wt genome or a significant drop in titer is inevitable. Foamy viruses have the largest genome among retroviruses and as a result, FV vectors have enough space to accommodate a little over 9 kb of exogenous DNA [44].

Finally, and aiming at practical applications, comes the issue of titers and storage. FV vectors can be concentrated by ultracentrifugation [44,45]. The codon optimization and expression design optimization of packaging and transfer vector plasmid sequences have allowed for the generation of high titer FV vector supernatants. These modifications rendered FV vectors as efficient as lentiviral vectors producing 10 × 10^7^ TU/mL of crude supernatant [44]. Finally, FV supernatants can be concentrated and purified in closed systems using affinity chromatography with POROS-Heparin columns, followed by Tangential Flow Filtration and ultracentrifugation [50] or size exclusion chromatography using CaptoCore columns followed by sepharose-heparin affinity chromatography and ultracentrifugation [51]. Both purification approaches are performed in closed systems compliant with cGMP and yield significantly pure preparations. Lastly, in regard to storage, although not reported in the literature per se, we have developed a freezing medium that allows repeated freeze/thaw cycles with 80–90% yields in vector titer.

## 3. Gene Marking Studies in Small Animals

Replication incompetent FV vectors were shown to transduce nearly every cell line including hematopoietic cell lines [33,34]. As the next leap forward, FV vectors were tested for their gene marking potential with murine HSCs after transplantation into lethally irradiated hosts [21]. The data showed that FV vectors could transduce murine HSC with a marking efficiency of about 50% and after transplantation there was sustained expression of the transgene for over 6 months. Similarly, when human HSCs (CD34+) cells were transduced and transplanted in ablated NOD/SCID animals, high levels of gene marking were observed across all lineages, indicating the transduction of a true human HSC [37]. In a direct head-to-head comparison between GV, LV and FV vectors with identical constructs, FV vectors performed as efficiently as the LV vectors, indicating FV vectors’ potential for clinical applications [52].

Another notable mention is that in close to 500 animals that we have transplanted, we never encountered an adverse outcome such as leukemia or lymphomaThese early observations on murine and human HSCs were the first indications that FV derived vectors were a relatively safe vector system that did not cause any harm in the HSC genomes and could thus be further tested in preclinical animal models as a therapeutic gene transfer vehicle.

## 4. Therapeutic Gene Transfer in Murine Preclinical Models

The testing ground for any gene therapy vector has traditionally been the genetic correction of β-thalassemia. Two such FV vectors have been developed and tested side-by-side. The expression cassette had the complete human β-globin gene under the control of the short native β-globin gene promoter with either an α-globin HS40 sequence, or a mini-LCR with the core sequences of the HS2 and HS3 regulatory elements from the β-globin LCR. Both vector viral stocks were used to transduce erythroleukemia lines, murine Lin- HSCs from normal and thalassemic mice and human CD34+ cells from β-thalassemia patients. The vectors had comparable efficiency in all settings, although the HS40 was marginally superior and more stable in vivo. In the thalassemic mouse model Hbbth3/+, the transplantation of FV-transduced HSCs with the HS40 vector resulted in 43% of peripheral blood expressing human β globin at 6 months post transplantation. This level of expression is adequate to establish a thalassemia carrier phenotype and a therapeutic effect [53].

Another monogenic recessive disorder amenable to treatment with gene therapy is chronic granulomatous disease (CGD) [54]; the X-linked form of the disease results from mutations in the CYBB gene that encodes the gp91phox, the larger subunit of the oxidase flavocytochrome b558. Patients with CGD lack production of microbicidal superoxide, resulting in recurrent infections and early deaths in childhood. An FV vector carrying the *gp91phox* gene under the control of a PGK or a MSCV-LTR promoter was tested for its ability to restore superoxide levels in transplanted animals. With an average of 41.5% chimerism, the transplanted animals displayed phagoburst activity that reached 40% of the wt animals, clearly indicating a robust therapeutic effect in the preclinical model [55,56]. Overall, superoxide production reached 70% of normal with low vector copy numbers per cell (<2), a finding that argues for copy number dependent expression and minimal cytotoxic effects. These levels of superoxide production are similar to what has been reported for lenti-based vectors [57].

FV vectors have also been used for the genetic correction of the Wiskott-Aldrich syndrome (WAS) mouse model [58]. WAS is an X-linked disorder characterized by eczema, immunodeficiency, and micro-thrombocytopenia resulting in bleeding tendency [59]. Uchiyama et al., used a WAS cDNA under the control of two different promoters (an endogenous and an A2UOCE-derived 631 bp fragment) and demonstrated the correction of the WAS phenotypic disorders. T-cell receptor-mediated responses, B-cell migration, platelet adhesion, and podosome formation in dendritic cells (DCs) were restored at levels that can translate into a therapeutic effect. In addition, they showed improvement in gene transfer rates with repeated transduction cycles and confirmed earlier integration site analyses. The IS distribution showed FV vectors landing within transcriptional units at a frequency of 22%–44%, versus 46%–72% for a similar WAS protein-expressing Lenti vector and a low tendency for integration near oncogenes (<5%). As the authors state, the combination of complete phenotypic restoration with low copy numbers (<2) and a relatively safe IS profile, make the WAS protein-expressing FV vectors attractive for clinical applications.

One of the first attempts of gene therapy in humans targeted the SCID-X1 disorder with retroviral vectors. The attempt was considered a success [8] but it was also an alarm on the side effects that should be addressed before the wide application of gene therapy [10]. The disease was targeted with an FV vector to test whether FVs could be an alternative vector system and to test whether the integration sites in T cells were relatively “safer” when FVs were used to transfer the γc gene [60]. In the ED40515 (γc-) T cell line, the FV vector integration sites were located in close proximity to the transcriptional start sites in 13% of integration events relative to 25% with the retroviral vectors. In addition, in the 100 IS analyzed, there were none located within oncogenes, as opposed to three, present in the retrovirally-transduced cells. Finally, animals transplanted with FV-corrected HSC cells, recovered their T and B cell counts and their serum levels of IgM, IgG, and IgA.

A relative problem with HSC gene transfer applications, specifically where in vivo selective advantage of transduced cells does not apply, has been the low gene transfer rates. A potential solution to this problem could be the use of pharmacologic agents that could selectively eliminate non-transduced cells. In an attempt to test whether resting HSC can be transduced and selected in vivo, investigators used the MGMT(P140K) DNA repair protein in an FV backbone. Transplanted mice were treated at 4 weeks post Bone Marrow Transplantation (BMT), with sub-myeloablative conditioning with O6-BG (O-6-Benzylguanine) and BCNU(bis-chloroethylnitrosourea) at different doses and analyzed 6 months later, the mock-transduced animals had undetectable levels of MGMT expression as compared to 55% of positive animals in the FV transduced group [61]. These results confirm the potential of FV vectors to transduce relatively resting HSC cells at low Multiplicity Of Infection (MOIs) and to selectively enhance their presence in the peripheral blood with pharmacologic manipulations. This approach was also used in the design of FV vectors that could block HIV replication. A cassette, that carried three anti-HIV targets in the form of short hairpins (*tat*/*rev* at site I and site II and to human CCR5), was able to confer 4 log reductions in HIV replication assays [49]. FV vectors are particularly useful for anti-HIV gene therapy, since using Lenti-based vectors for RNAi delivery could be problematic since the anti-HIV sequences can jeopardize the vector packaging process [62]. Beyond therapeutic gene transfer targeting single gene disorders, FV vectors designed to deliver shRNA through PolIII promoters after HSC transfer and transplantation provided a significant long-term downregulation of target genes [63].

Overall, the potential of FV vectors to correct the genetic defect in monogenic disorders amenable to HSC gene addition has been well established in various disease settings. In addition, FV vectors perform as good as their LV counterpart and have not been linked to any adverse effects.

## 5. Large Animal Preclinical Models

Large animal models offer the potential to simulate conditions that are much closer to the clinical setting as compared to the murine preclinical models. FV vectors have been used to treat two such conditions, the leukocyte adhesion deficiency (LAD) and the pyruvate kinase deficiency (PKD).

LAD is a stem cells disorder that affects white blood cell migration in response to chemotactic signals and as a result, affects immune responses. Patients suffer from bacterial and fungal infections that most commonly occur on the skin and mucous membranes. The defects affect the leukocyte integrin ITGB2 gene (CD18) that prevents the expression of the CD11/CD18 adhesion complex on the cell surface [64]. The canine form of the disease (CLAD) recapitulates the severe deficiency phenotype of LAD-1 in children [65].

Canine HSCs were transduced with an FV vector expressing normal CD18 cDNA and were transplanted to affected dogs [66]. After a year, the dogs had their WBC counts restored to normal and survived without antibiotics, both signs of a functional cure of their disease. The observed long-term (24 months) lymphocyte marking rates of 5–10% are compatible with normal life expectancy. This result was achieved with a single overnight exposure to the vector and a nonmyeloablative conditioning regimen. Furthermore, the copy number per cell was low and in the order of 0.83-1.25 provirus copies per diploid cell.

In the canine setting, FV vectors have also been used to treat a Basenji pyruvate kinase deficient dog model. Pyruvate kinase deficiency causes severe hemolytic anemia, which is potentially lethal [67]. Since PKD does not provide survival advantage to the successfully transduced cells, the vector was enhanced by co-expressing the mutant MGMTP140K that provides resistance to O6-benzylguanine and BCNU, potent inhibitors of the wt MGMTP protein that is expressed in all normal tissues. At 100 days post HSC transplantation with transduced cells, the gene marking rates were 3.5% for granulocytes and 0.4% for lymphocytes. After three rounds of treatment with O6BG and BCNU, gene marking raised to 33% in granulocytes and 5.5% in lymphocytes [68]. This also translated to the correction of the phenotypic disorder, as evidenced by the normalization of LDH (an indicator of hemolysis) and the achievement of transfusion independence.

Finally, it has to be mentioned that FV vectors are resistant to lysis by human serum, a property that has sparked interest for direct in vivo delivery of the vectors, avoiding all ex vivo manipulations of HSCs. This has been tried with the X-SCID canine model [69]. A total of five newborn SCID-X1 dogs received i.v. infusions of FV vector preparations with doses ranging from 4.0-8.4 x10^8 particles. The functional outcomes showed marginal immune reconstitution. The dogs did not survive past one year and succumbed to infections. In regard to off target viral integrations, two such sites were recorded: one in the gut, another (potential) at the virus infusion site and none in the germ cells. An improved protocol was later implemented from the same group that included stem cell mobilization with G-CSF/AMD3100 prior to FV vector delivery and the substitution of the EF1a promoter with that of the *pgk* gene in the FV vector [59]. The data argue for the faster recovery of T cell numbers and a broad TcR repertoire, and are clinically relevant when considering the overall survival that climbed to 2.5 years as compared to 330 days in the previous study. On the issue of safety, a major concern when a vector with broad cell tropism is tested, two conclusions emerged from this study: (i) the vast majority of integration sites were shared between tissues and blood indicating blood contamination and (ii) the gonads had the smallest number of integration events (37 and 56 for ovaries and testes, respectively, as compared to 766 and 469 in blood) with none of them being unique except for one integration site in the ovaries that may have been derived from non-germ cells.

## 6. Conclusions

Foamy virus vectors have been extensively tested in marking and in gene therapy studies with small and big preclinical animal models. The gene therapy trials featured in this review are summarized in Table 2. Their non-pathogenic nature is an attractive feature for clinical applications. This is further supported by all the positive outcomes that the gene therapy community has communicated from two decades of testing.It is therefore strange that these vectors have not found their way into the clinic. It seems from the relative literature that most manufacturing issues have been overcome and what is missing is the interest of pharmaceutical companies to develop it as a product. In the meantime, the gene therapy world has been dominated by LV vectors that are offered as pharmaceutical products often at extraordinary prices [70]. FVs with the relative ease of production, concentration, and purification could become a poor man’s Ferrari for nations and insurance systems that cannot afford million-dollar price tags for a relatively simple and one-off treatment.

## Figures and Tables

**Table 1:** Features of Retroviral gene delivery systems.

Vector System	Lenti-	Gammaretro-	Foamy-
Transgene Capacity (kb)	9 [26]	10 [27]	At least 9 [28]
Self-Inactivating (SIN) design	+ [29]	+ [30]	+ [31]
Generation	3rd	3rd	3rd
Presence of insulators in design	+	+	+
Pseudotyping	+	+	-
Cell cycle requirement			
cGMP complience	+	+	possible
Preferred integration sites in host genome	Active transcriptional units [11]	Transcriptional start sites and CpG islands [11,32]	Constitutively lamina associated regions (cLAD) and less often CpGs [15]

**Table 2:** Gene therapy trials with FV-derived vectors.

Disease	Animal Model	FV Vector Systems	Promoter	Transgene	Target Cells	Method of Application	Outcome	Reference
**β-thalassemia**	β-Thal3 mice	3rd	Hu-α-globin HS40-short hu-β-globin	hu-β-globin	Lin- BM HSCs	Ex vivo	Conversion to thalassemia carrier phenotype	[53]
		3rd	Hu-β-globin HS2-HS3 LCR-short hu-β-globin					
**Chronic Granulomatus Disease (CGD)**	B6.129S-Cybb^tm1Din^/J mice	3rd	PGK	c-o hu-gp91phox	Lin-BM HSCs	Ex vivo	Complete phenotypic restoration	[55]
		3rd	MSCV-LTR	c-o hu-gp91phox IRES.EGFP				
**Wiskott-Aldrich Syndrome (WAS)**	WAS KO mice	3rd	Native promoter	hu-was	Lin- BM HSCs	Ex vivo	Complete phenotypic restoration	[58]
		3rd	UCO631					
**SCID-X1**	NOD/SCID γc KO mice	3rd	UCO631	Human γc gene (IL2RG)	Lin- BM HSCs	Ex vivo	Reconstitution of T and B cells. No NK correction	[60]
**SCID-X1**	SCID-X1 dogs	3rd	EF1a (intronless)	GFP.T2A.hIL2RG	i.v. infusion	In vivo	Partial lymphocyte reconstitution	[69]
					i.v. infusion in HSC mobilized animals		Lymphocyte reconstitution. Successful treatment	[59]
**SCID-X1**	SCID-X1 dogs	3rd	PGK	GFP.T2A.hIL2RG	i.v. infusion in mobilized HSC	In vivo	Lymphocyte reconstitution. Phenotypic correction	
**Leukocyte adhesion deficiency**	CLAD dogs	3rd	MSCV	hu-CD18	BM derived CD34+ cells	Ex vivo	Phenotypic correction	[66]
**Pyruvate Kinase deficiency**	Basenji Dog PKD	3rd	PGK	SFFVMGMT.T2AEGFP.PGK.cPK	Mobilized CD34+ HSCs	Ex vivo	Phenotypic correction	[67]

## References

[1] Seita J., Weissman I.L. (2010). Hematopoietic stem cell: Self-renewal versus differentiation. Wiley Interdiscip. Rev. Syst. Biol. Med..

[2] Lucas D. (2017). The Bone Marrow Microenvironment for Hematopoietic Stem Cells. Adv. Exp. Med. Biol..

[3] Heimfeld S. (2003). Bone marrow transplantation: How important is CD34 cell dose in HLA-identical stem cell transplantation?. Leukemia.

[4] Lucarelli G., Isgro A., Sodani P., Gaziev J. (2012). Hematopoietic stem cell transplantation in thalassemia and sickle cell anemia. Cold Spring Harb. Perspect. Med..

[5] Dunbar C.E., High K.A., Joung J.K., Kohn D.B., Ozawa K., Sadelain M. (2018). Gene therapy comes of age. Science.

[6] Papapetrou E.P., Ziros P.G., Micheva I.D., Zoumbos N.C., Athanassiadou A. (2006). Gene transfer into human hematopoietic progenitor cells with an episomal vector carrying an S/MAR element. Gene Ther..

[7] Khan A.S., Bodem J., Buseyne F., Gessain A., Johnson W., Kuhn J.H., Kuzmak J., Lindemann D., Linial M.L., Lochelt M. (2019). Corrigendum to “Spumaretroviruses: Updated taxonomy and nomenclature” [Virology 516 (2018) 158-164]. Virology.

[8] Cavazzana-Calvo M., Hacein-Bey S., de Saint Basile G., Gross F., Yvon E., Nusbaum P., Selz F., Hue C., Certain S., Casanova J.L. (2000). Gene therapy of human severe combined immunodeficiency (SCID)-X1 disease. Science.

[9] Aiuti A., Cattaneo F., Galimberti S., Benninghoff U., Cassani B., Callegaro L., Scaramuzza S., Andolfi G., Mirolo M., Brigida I. (2009). Gene therapy for immunodeficiency due to adenosine deaminase deficiency. N. Engl. J. Med..

[10] Hacein-Bey-Abina S., Garrigue A., Wang G.P., Soulier J., Lim A., Morillon E., Clappier E., Caccavelli L., Delabesse E., Beldjord K. (2008). Insertional oncogenesis in 4 patients after retrovirus-mediated gene therapy of SCID-X1. J. Clin. Investig..

[11] Lesbats P., Engelman A.N., Cherepanov P. (2016). Retroviral DNA Integration. Chem. Rev..

[12] Cattoglio C., Facchini G., Sartori D., Antonelli A., Miccio A., Cassani B., Schmidt M., von Kalle C., Howe S., Thrasher A.J. (2007). Hot spots of retroviral integration in human CD34+ hematopoietic cells. Blood.

[13] Trobridge G.D., Miller D.G., Jacobs M.A., Allen J.M., Kiem H.P., Kaul R., Russell D.W. (2006). Foamy virus vector integration sites in normal human cells. Proc. Natl. Acad. Sci. USA.

[14] Nowrouzi A., Dittrich M., Klanke C., Heinkelein M., Rammling M., Dandekar T., von Kalle C., Rethwilm A. (2006). Genome-wide mapping of foamy virus vector integrations into a human cell line. J. Gen. Virol..

[15] Lesbats P., Serrao E., Maskell D.P., Pye V.E., O’Reilly N., Lindemann D., Engelman A.N., Cherepanov P. (2017). Structural basis for spumavirus GAG tethering to chromatin. Proc. Natl. Acad. Sci. USA.

[16] Challita P.M., Kohn D.B. (1994). Lack of expression from a retroviral vector after transduction of murine hematopoietic stem cells is associated with methylation in vivo. Proc. Natl. Acad. Sci. USA.

[17] Ellis J. (2005). Silencing and variegation of gammaretrovirus and lentivirus vectors. Hum. Gene Ther..

[18] Li C.L., Emery D.W. (2008). The cHS4 chromatin insulator reduces gammaretroviral vector silencing by epigenetic modifications of integrated provirus. Gene Ther..

[19] McInerney J.M., Nawrocki J.R., Lowrey C.H. (2000). Long-term silencing of retroviral vectors is resistant to reversal by trichostatin A and 5-azacytidine. Gene Ther..

[20] Miyoshi H., Smith K.A., Mosier D.E., Verma I.M., Torbett B.E. (1999). Transduction of human CD34+ cells that mediate long-term engraftment of NOD/SCID mice by HIV vectors. Science.

[21] Vassilopoulos G., Trobridge G., Josephson N.C., Russell D.W. (2001). Gene transfer into murine hematopoietic stem cells with helper-free foamy virus vectors. Blood.

[22] Lindemann D., Rethwilm A. (2011). Foamy virus biology and its application for vector development. Viruses.

[23] Vassilopoulos G., Rethwilm A. (2008). The usefulness of a perfect parasite. Gene Ther..

[24] Liu W., Lei J., Liu Y., Lukic D.S., Rathe A.M., Bao Q., Kehl T., Bleiholder A., Hechler T., Lochelt M. (2013). Feline foamy virus-based vectors: Advantages of an authentic animal model. Viruses.

[25] Ledesma-Feliciano C., Hagen S., Troyer R., Zheng X., Musselman E., Slavkovic Lukic D., Franke A.M., Maeda D., Zielonka J., Munk C. (2018). Replacement of feline foamy virus bet by feline immunodeficiency virus vif yields replicative virus with novel vaccine candidate potential. Retrovirology.

[26] Lamsfus-Calle A., Daniel-Moreno A., Urena-Bailen G., Raju J., Antony J.S., Handgretinger R., Mezger M. (2019). Hematopoietic stem cell gene therapy: The optimal use of lentivirus and gene editing approaches. Blood Rev..

[27] Maetzig T., Galla M., Baum C., Schambach A. (2011). Gammaretroviral vectors: Biology, technology and application. Viruses.

[28] Trobridge G.D. (2009). Foamy virus vectors for gene transfer. Expert Opin. Biol. Ther..

[29] Miyoshi H., Blomer U., Takahashi M., Gage F.H., Verma I.M. (1998). Development of a self-inactivating lentivirus vector. J. Virol..

[30] Thornhill S.I., Schambach A., Howe S.J., Ulaganathan M., Grassman E., Williams D., Schiedlmeier B., Sebire N.J., Gaspar H.B., Kinnon C. (2008). Self-inactivating gammaretroviral vectors for gene therapy of X-linked severe combined immunodeficiency. Mol. Ther..

[31] Goodman M.A., Arumugam P., Pillis D.M., Loberg A., Nasimuzzaman M., Lynn D., van der Loo J.C.M., Dexheimer P.J., Keddache M., Bauer T.R. (2018). Foamy Virus Vector Carries a Strong Insulator in Its Long Terminal Repeat Which Reduces Its Genotoxic Potential. J. Virol..

[32] Roth S.L., Malani N., Bushman F.D. (2011). Gammaretroviral integration into nucleosomal target DNA in vivo. J. Virol..

[33] Russell D.W., Miller A.D. (1996). Foamy virus vectors. J. Virol..

[34] Hirata R.K., Miller A.D., Andrews R.G., Russell D.W. (1996). Transduction of hematopoietic cells by foamy virus vectors. Blood.

[35] Plochmann K., Horn A., Gschmack E., Armbruster N., Krieg J., Wiktorowicz T., Weber C., Stirnnagel K., Lindemann D., Rethwilm A. (2012). Heparan sulfate is an attachment factor for foamy virus entry. J. Virol..

[36] Nasimuzzaman M., Persons D.A. (2012). Cell Membrane-associated heparan sulfate is a receptor for prototype foamy virus in human, monkey, and rodent cells. Mol. Ther..

[37] Josephson N.C., Vassilopoulos G., Trobridge G.D., Priestley G.V., Wood B.L., Papayannopoulou T., Russell D.W. (2002). Transduction of human NOD/SCID-repopulating cells with both lymphoid and myeloid potential by foamy virus vectors. Proc. Natl. Acad. Sci. USA.

[38] Trobridge G., Russell D.W. (2004). Cell cycle requirements for transduction by foamy virus vectors compared to those of oncovirus and lentivirus vectors. J. Virol..

[39] Lehmann-Che J., Renault N., Giron M.L., Roingeard P., Clave E., Tobaly-Tapiero J., Bittoun P., Toubert A., de The H., Saib A. (2007). Centrosomal latency of incoming foamy viruses in resting cells. PLoS Pathog..

[40] Hendrie P.C., Huo Y., Stolitenko R.B., Russell D.W. (2008). A rapid and quantitative assay for measuring neighboring gene activation by vector proviruses. Mol. Ther..

[41] Ong C.T., Corces V.G. (2014). CTCF: An architectural protein bridging genome topology and function. Nat. Rev. Genet..

[42] Heneine W., Switzer W.M., Sandstrom P., Brown J., Vedapuri S., Schable C.A., Khan A.S., Lerche N.W., Schweizer M., Neumann-Haefelin D. (1998). Identification of a human population infected with simian foamy viruses. Nat. Med..

[43] Switzer W.M., Bhullar V., Shanmugam V., Cong M.E., Parekh B., Lerche N.W., Yee J.L., Ely J.J., Boneva R., Chapman L.E. (2004). Frequent simian foamy virus infection in persons occupationally exposed to nonhuman primates. J. Virol..

[44] Trobridge G., Josephson N., Vassilopoulos G., Mac J., Russell D.W. (2002). Improved foamy virus vectors with minimal viral sequences. Mol. Ther..

[45] Nasimuzzaman M., Kim Y.S., Wang Y.D., Persons D.A. (2014). High-titer foamy virus vector transduction and integration sites of human CD34+ cell-derived SCID-repopulating cells. Mol. Ther. Methods Clin. Dev..

[46] Bock M., Heinkelein M., Lindemann D., Rethwilm A. (1998). Cells expressing the human foamy virus (HFV) accessory Bet protein are resistant to productive HFV superinfection. Virology.

[47] Heinkelein M., Dressler M., Jarmy G., Rammling M., Imrich H., Thurow J., Lindemann D., Rethwilm A. (2002). Improved primate foamy virus vectors and packaging constructs. J. Virol..

[48] Lochelt M., Zentgraf H., Flugel R.M. (1991). Construction of an infectious DNA clone of the full-length human spumaretrovirus genome and mutagenesis of the bel 1 gene. Virology.

[49] Kiem H.P., Wu R.A., Sun G., von Laer D., Rossi J.J., Trobridge G.D. (2010). Foamy combinatorial anti-HIV vectors with MGMTP140K potently inhibit HIV-1 and SHIV replication and mediate selection in vivo. Gene Ther..

[50] Nasimuzzaman M., Lynn D., Ernst R., Beuerlein M., Smith R.H., Shrestha A., Cross S., Link K., Lutzko C., Nordling D. (2016). Production and purification of high-titer foamy virus vector for the treatment of leukocyte adhesion deficiency. Mol. Ther. Methods Clin. Dev..

[51] Spannaus R., Miller C., Lindemann D., Bodem J. (2017). Purification of foamy viral particles. Virology.

[52] Leurs C., Jansen M., Pollok K.E., Heinkelein M., Schmidt M., Wissler M., Lindemann D., Von Kalle C., Rethwilm A., Williams D.A. (2003). Comparison of three retroviral vector systems for transduction of nonobese diabetic/severe combined immunodeficiency mice repopulating human CD34+ cord blood cells. Hum. Gene Ther..

[53] Morianos I., Siapati E.K., Pongas G., Vassilopoulos G. (2012). Comparative analysis of FV vectors with human alpha- or beta-globin gene regulatory elements for the correction of beta-thalassemia. Gene Ther..

[54] Kang E.M., Malech H.L. (2012). Gene therapy for chronic granulomatous disease. Methods Enzymol..

[55] Chatziandreou I., Siapati E.K., Vassilopoulos G. (2011). Genetic correction of X-linked chronic granulomatous disease with novel foamy virus vectors. Exp. Hematol..

[56] Marciano B.E., Zerbe C.S., Falcone E.L., Ding L., DeRavin S.S., Daub J., Kreuzburg S., Yockey L., Hunsberger S., Foruraghi L. (2018). X-linked carriers of chronic granulomatous disease: Illness, lyonization, and stability. J. Allergy Clin. Immunol..

[57] Chiriaco M., Farinelli G., Capo V., Zonari E., Scaramuzza S., Di Matteo G., Sergi L.S., Migliavacca M., Hernandez R.J., Bombelli F. (2014). Dual-regulated lentiviral vector for gene therapy of X-linked chronic granulomatosis. Mol. Ther..

[58] Uchiyama T., Adriani M., Jagadeesh G.J., Paine A., Candotti F. (2012). Foamy virus vector-mediated gene correction of a mouse model of Wiskott-Aldrich syndrome. Mol. Ther..

[59] Humbert O., Chan F., Rajawat Y.S., Torgerson T.R., Burtner C.R., Hubbard N.W., Humphrys D., Norgaard Z.K., O’Donnell P., Adair J.E. (2018). Rapid immune reconstitution of SCID-X1 canines after G-CSF/AMD3100 mobilization and in vivo gene therapy. Blood Adv..

[60] Horino S., Uchiyama T., So T., Nagashima H., Sun S.L., Sato M., Asao A., Haji Y., Sasahara Y., Candotti F. (2013). Gene therapy model of X-linked severe combined immunodeficiency using a modified foamy virus vector. PLoS ONE.

[61] Cai S., Ernstberger A., Wang H., Bailey B.J., Hartwell J.R., Sinn A.L., Eckermann O., Linka Y., Goebel W.S., Hanenberg H. (2008). In vivo selection of hematopoietic stem cells transduced at a low multiplicity-of-infection with a foamy viral MGMT (P140K) vector. Exp. Hematol..

[62] Olszko M.E., Trobridge G.D. (2013). Foamy virus vectors for HIV gene therapy. Viruses.

[63] Papadaki M., Siapati E.K., Vassilopoulos G. (2011). A foamy virus vector system for stable and efficient RNAi expression in mammalian cells. Hum. Gene Ther..

[64] Leukocyte Adhesion Deficiency Type 1. https://ghr.nlm.nih.gov/condition/leukocyte-adhesion-deficiency-type-1.

[65] Kijas J.M., Bauer T.R., Gafvert S., Marklund S., Trowald-Wigh G., Johannisson A., Hedhammar A., Binns M., Juneja R.K., Hickstein D.D. (1999). A missense mutation in the beta-2 integrin gene (ITGB2) causes canine leukocyte adhesion deficiency. Genomics.

[66] Bauer T.R., Allen J.M., Hai M., Tuschong L.M., Khan I.F., Olson E.M., Adler R.L., Burkholder T.H., Gu Y.C., Russell D.W. (2008). Successful treatment of canine leukocyte adhesion deficiency by foamy virus vectors. Nat. Med..

[67] Takegawa S., Fujii H., Miwa S. (1983). Change of pyruvate kinase isozymes from M2- to L-type during development of the red cell. Br. J. Haematol..

[68] Trobridge G.D., Beard B.C., Wu R.A., Ironside C., Malik P., Kiem H.P. (2012). Stem cell selection in vivo using foamy vectors cures canine pyruvate kinase deficiency. PLoS ONE.

[69] Burtner C.R., Beard B.C., Kennedy D.R., Wohlfahrt M.E., Adair J.E., Trobridge G.D., Scharenberg A.M., Torgerson T.R., Rawlings D.J., Felsburg P.J. (2014). Intravenous injection of a foamy virus vector to correct canine SCID-X1. Blood.

[70] Macaulay R. How CAR-T Cell and Gene Therapies Are Redefining the Traditional Pharmaceutical Pricing and Reimbursement Model. https://www.parexel.com/news-events-resources/blog/how-car-t-cell-and-gene-therapies-are-redefining-traditional-pharmaceutical-pricing-and-reimbursement-model.

